# Skin color reporting in squamous cell carcinoma-related randomized controlled trials in top dermatology journals: a systematic review

**DOI:** 10.1007/s00403-024-02843-2

**Published:** 2024-03-30

**Authors:** Natasha L. Salmen, Klarens Menage, Anthony N. Baumann, Deven P. Curtis, Robert T. Brodell

**Affiliations:** 1 Northeast Ohio Medical University, Rootstown, OH United States; 2 Department of Pathology, University of Mississippi Medical Center, Jackson, MS USA

**Keywords:** Skin of color, Squamous cell carcinoma, Fitzpatrick scale

## Abstract

The objectives are to determine the frequency that skin color is reported in randomized controlled trials (RCTs) involving squamous cell carcinoma (SCC) detection and treatment in leading dermatology journals. A systematic review of RCTs involving SCC was conducted among the top ten most impactful dermatology journals from inception to July 10th, 2023. Studies were included if they reviewed the treatment, prevention, or detection of SCC, involved patients directly and were classified as traditional RCTs. Studies were considered positive for reporting SOC if there was any demographic data in the methods or results of the following terms: Fitzpatrick scale, race, ethnicity, sunburn tendency, or skin of color. Of the 39 studies which were identified, 23 reported data related to skin color data (59.0%). White individuals were the most reported in these studies (56.5%). Subgroup analysis was conducted, and no statistical significance was found for study location, year of publication, or funding source. Skin color impacts skin cancer detection, predominant location of tumors, and recurrence. Less than 60% of high-quality RCTs related to SCC in top global dermatology journals included skin color among the demographic traits of study participants. Subgroup analysis demonstrated no improvement in reporting over the past 2 decades. Further research is needed to understand the reason for low skin color reporting rates among SCC-related RCTs and the impact this has on society.

**Supplementary Information:**

The online version contains supplementary material available at 10.1007/s00403-024-02843-2.

## Introduction

Squamous cell carcinoma (SCC) is the second most common type of keratinocyte skin cancer worldwide [1]. It accounts for about 20% of all skin cancers and has an estimated annual incidence of 700,000 cases in the US [1, 2]. Furthermore, the incidence of SCC has increased over 200% in the last 3 decades. Estimates suggest it will continue to increase at a rate of 23% for males and 29% for females in the next few years [3, 4].

SCC originates from the malignant proliferation of keratinocytes in the epidermis and adnexal structures such as eccrine glands and pilosebaceous units [4]. The major modifiable risk factor contributing to SCC is ultraviolet (UV) exposure from the sun and tanning parlors. Immunosuppression and fair skin are also significant risk factors. Melanin is a broadband UV absorbent which protects against the carcinogenic effects of UV light, thus decreasing the risk of skin cancer [5, 6].

SCC is the second most common type of skin cancer in Caucasian, Hispanic and Asian individuals [7]. However, SCC is the most common type of skin cancer in African Americans; although, the incidence is low compared to individuals with light skin. Interestingly, people with darker skin tones develop SCC in areas that are not exposed to the sun. Patients with albinism develop SOC in areas similar to white individuals [5]. The clinical presentation also differs in patients with SOC. SCC in Caucasians presents with superficial, scaling lesions with a raised base. In SOC, SCC presents as a non-healing erosion or ulceration that may bleed and can also develop following trauma-induced scarring or thermal burns [8, 9]. In both light and dark skin tones, SCC is often preceded by the development of actinic keratosis (AK) [5].

Since the clinical presentation and risk factors for SCC differ between skin colors, it is critical for studies to include patients from all races and ethnicities. The frequency of reporting skin color in randomized controlled trials (RCTs) involving SCC has not previously been reported. The purpose of this systematic review is to determine the rate of skin color/tone, race or ethnicity reporting in RCTs involving SCC in the top ten most impactful international dermatology journals.

## Methods

### Study design

**Fig. 1:**
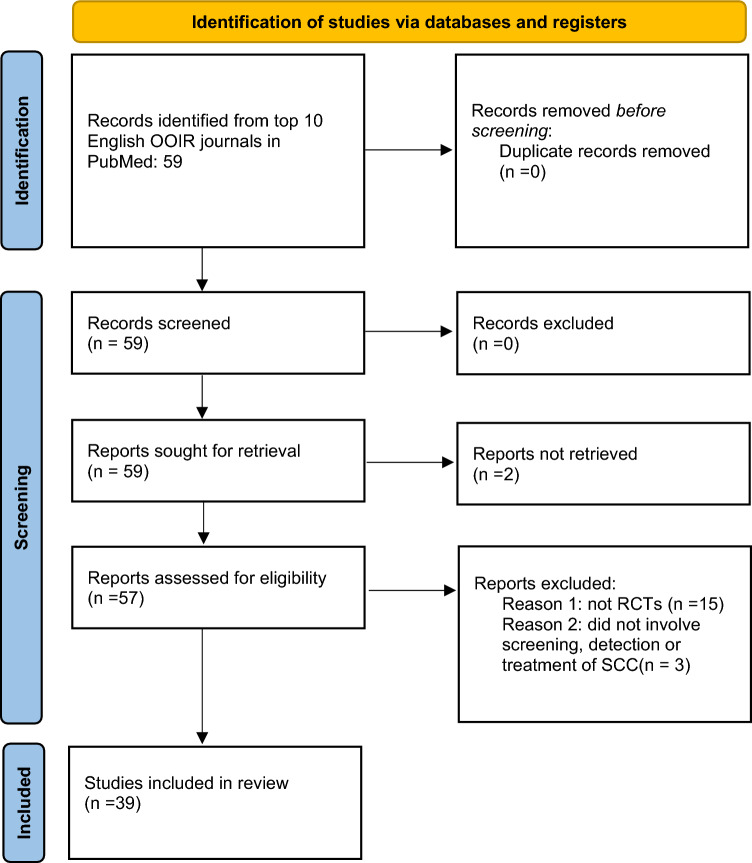
Preferred Reporting Items for Systematic Review and Meta-Analyses (PRISMA) diagram

A systematic review of RCTs involving SCC was conducted up to July 10, 2023. Only the top ten dermatology journals in the world, written in English, as determined by impact factor, were included in the search. These journals were selected from a rank list by the Observatory of International Research (see Table 1). The 9th ranked journal of the list was the Journal der Deutschen Dermatologischen Gesellschaft and was written in German. Thus, the 11th journal, Dermatitis, was included instead as this was written in English. PubMed was used to conduct the search. Search terms included the abbreviations for the journals, as seen in Table 1, as well as “squamous cell carcinoma” and were filtered by RCTs. This study follows the guidelines of the Preferred Reporting Items for Systematic Review and Meta-Analyses (PRISMA), as seen in Fig. 1.

**Table 1:** List of the top ten most impactful dermatology journals from the Observatory of International Research, July 2023

Journal	Impact factor	PubMed term
Journal of the American Academy of Dermatology	15.49	J Am Acad Dermatol
JAMA Dermatology	11.82	JAMA Dermatology
British Journal of Dermatology	11.11	Br J Dermatol
Journal of the European Academy of Dermatology and Venerology	9.23	J Eur Acad Dermatol Venereol
Journal of Investigative Dermatology	7.59	J Invest Dermatol
Contact Dermatitis	6.42	Contact Dermatitis
American Journal of Clinical Dermatology	6.23	Am J Clin Dermatol
Journal of Dermatological Science	5.41	J Dermatol Sci
Dermatology	5.2	Dermatology
Dermatitis	5.19	Dermatitis

### Inclusion and exclusion criteria

To meet inclusion criteria all selected RCT articles: assessed SCC (treatment, detection, or prevention), involved human patients, and were written in English. Articles were excluded if: they did not involve patients (such as RCTs evaluating educational programs) or if the full text was unavailable.

### Study categories

Subgroup analysis was conducted based on categories including: year groups (prior to 2010 vs. 2010 and later), funding sources (industry vs. non-industry), and study locations (conducted in the United States vs. outside of the United States).

### Primary outcome measures

The primary outcome: report of any term denoting skin color including: race, ethnicity, Fitzpatrick rating, sunburn tendency, melanin content, or pigmentation of the study participants anywhere as reflected in data and results. The primary outcome measure was then stratified by year of publication, funding sources, and study location. Articles that did report on the skin color and/or race were assessed for the type of skin color and/or race that may have been reported for all participants in the study.

### Article sorting process

After collecting the initial articles, all were imported into Rayyan.ai, an online software for systematic reviews, for screening and organization. One author (DC) manually removed duplicates.

### Data extraction

Included articles were imported into a spreadsheet. Two coauthors (NS, DC) independently screened the articles to determine if they met inclusion criteria. Data were extracted and organized into the following template categories:

- Journal
- Title
- Author
- Year of publication
- Full text availability
- Study type
- Involves treatment/prevention/detection of SCC in humans
- Include or exclude
- Reason if excluded
- Mentions skin color/race/ethnicity
- Reports race/skin color/ethnicity
- Terms used to describe skin
- Funding source
- Primary study location

### Statistical analysis

The Statistical Package for the Social Sciences (SPSS) version 29.0 (Armonk, NY: IBM Corp) was utilized for statistical analysis in this study. Descriptive statistics, such as frequency counts, were used to present the data when appropriate. Fisher’s exact test was used to compare categorical groups with data due to the small sample size. All *p* values (two-sided) less than 0.05 were considered statistically significant.

## Results

### Initial search results

A total of 39 RCTs met final inclusion criteria from 59 articles initially retrieved (Fig. 1, PRISMA).

### Article demographics

Of the total 39 RCTs included in this systematic review, 23 RCTs (59.0%) reported on skin color and 16 RCTs (41.0%) did not report on skin color and/or race. Of the 23 RCTs that did report on skin color, 17 RCTs (73.9%) used the Fitzpatrick scale or skin type (graded from I to V in the included studies). For specified race, white/Caucasian/northern European was the most common (56.5%; *n* = 13 studies reported) with black (8.7%; *n* = 2 studies reported) being the second most common. Refer to Table 2 for more detail on the skin color and/or race reported on each of the included studies.

**Table 2:** Specifics of skin color and/or race reported in included articles

White/Caucasian/northern European	*n* = 13, 56.5%
Black	*n* = 2, 8.7%
Hispanic/brown	*n* = 3, 13%
Asian	*n* = 2, 8.7%
Other (Native American, Middle Eastern,…etc.)	*n* = 3, 13%

### Study location on reporting

Of the total 39 RCTs included in this systematic review, 9 (23.1%) were conducted in the United States whereas 30 RCTs (76.9%) were conducted outside of the United States. There was no statistically significant difference in the proportion of RCTs that reported on skin color and/or race between RCTs conducted in the United States (55.6%; *n* = 5 cases out of 9 studies) as compared to RCTs conducted outside of the United States (60.0%; *n* = 18 cases out of 30 studies) (*p* = 1.000).

### Funding source on reporting

Of the total of 39 included RCTs, 11 (28.2%) received industry funding whereas 28 (71.8%) did not receive industry funding. There was no statistically significant difference in the proportion of RCTs that reported on skin color and/or race between RCTs that received industry funding (63.6%; *n* = 7 cases out of 11 studies) as compared to RCTs that did not receive industry funding (57.1%; *n* = 16 cases out of 28 studies) (*p* = 1.000). For all funding types for the included RCTs (*n* = 39 studies), 11 RCTs received industry funding (14.7%), 6 RCTs received grant funding (8.0%), 6 RCTs did not receive funding (8.0%), 13 studies had unknown funding sources (17.3%), and 3 RCTs (4.0) had other funding sources.

### Year of publication on reporting

14 (35.9%) of the 39 included RCTs were published prior to 2010 whereas 25 RCTs (64.1%) were published in 2010 or later. There was no statistically significant difference in the proportion of RCTs that reported on skin color and/or race between RCTs published prior to 2010 (57.1%; *n* = 8 cases out of 14 studies) as compared to RCTs published in 2010 or later (60%; *n* = 15 cases out of 25 studies) (*p* = 1.000).

## Discussion

The findings of this systematic review suggest that skin color is reported in RCTs involving SCC management, detection, and treatment a little over half of the articles (59.0%). A firstness study, confirmed that there has not been another systematic review conducted to assess whether skin color is reported in SCC-related RCTs. A gap of knowledge in populations, can result in poor patient outcomes when individual patients are treated. Any disparity related to prevention or treatment should be addressed in minority populations.

Of the 23 studies that reported skin color in this systematic review, 56.5% of participants were white/Caucasian or Northern European. Of these 23 studies, 7 (30.4%) included only white/Caucasian patients in their RCTs. Since SCC lesions can have varying presentations in types of skin color, it is crucial to include patients with skin of color in these RCTs and emphasize these differences. Similarly, 3 of the 23 studies (13.0%) categorized various ethnic minorities into one group. For example, one study included many ethnic groups; however, Mediterranean, Middle Eastern, Hispanic, Asian, Latin American, and Native American were classified into the same category within their study on predictors of SCC [10]. The skin color of these groups varies significantly.

On the other hand, the Fitzpatrick scale was utilized in 73.9% of the studies that reported skin color. This scale can help distinguish between varying skin tones. Most of these studies included primarily patients with a skin tone between I and III on the Fitzpatrick scale (light skin coloration). One study characterized skin type into four different groups, including moderately dark, slightly dark, fair and pale, rather than race or ethnicity, to discuss a topical treatment for AK [11]. The Fitzpatrick scale was also utilized to provide more insight into the skin tones of the patients. With this information, only three patients in this study were identified with grade IV skin on the Fitzpatrick scale. Since patients with fairer skin are more susceptible to UV radiation and ultimately AK, it is logical that these patients would be easier to recruit [12].

There was no significant difference between the studies reporting skin coloration within the United States (23.1%) compared to those conducted outside of the United States (71.8%). As of July 1, 2023, the U.S. is composed of 58.9% of whites, non-Hispanic or Latino [13]. This leaves 41.1% of the population as non-white, and a substantial portion of the data in these studies may not be applicable to their treatment.

There was also no significant difference in reporting skin color in subgroup analysis between years of publication prior to 2010 and after 2010. Skin color reporting in RCTs related to SCC has not increased over the years, which continues the disparity of information regarding the diagnosis, prevention, and treatment of SCC in non-white individuals. Additionally, socioeconomic factors may impact skin cancer detection and treatment for individuals with SOC, as well as affect their participation in research studies. These individuals might face barriers in accessing the necessary care, such as lack of healthcare insurance and lack of transportation [9]. Education is another important factor that may hinder people with SOC from seeking care. There are misconceptions about the risk of skin cancer in these individuals, which may discourage them from seeking care [14]. Similarly, obstacles may exist for some minority individuals that prevent them from engaging in clinical trials, such as lack of transportation, health literacy, inability to miss work, distance to clinical site, eligibility requirements, and distrust in the medical community [15]. Given the history the U.S. has involving research on people with SOC, there is now a hesitancy for these individuals to willingly participate in studies. To enhance the care for patients with SOC, further research is needed that tailors their specific needs.

## Conclusion

Skin color affects the detection, prevention, diagnosis, and treatment of skin cancer. This systematic review assessed RCTs focused on SCC in the top eleven global dermatology journals. Fewer than 60% of these studies incorporated skin color in their demographics reports of study participants. Although SCC is not the most deadly or the most common type of skin cancer, it is still a neoplasm that affects individuals of all skin colors. Since SCC can have varying clinical presentations and risk factors in various races, it is critical for researchers to incorporate skin color demographics within their studies. Subgroup analysis demonstrated no statistical significance dependent upon study location, funding source or year of publication. Additionally, subgroup analysis found no improvement in reporting skin color over the past 2 decades. Further research is necessary to determine the reasons for these findings and assess the impact of low skin color reporting rates among SCC-related RCTs.

### Supplementary Information

Below is the link to the electronic supplementary material.

Supplementary file1 (DOCX 21 KB)

## Data Availability

No datasets were generated or analysed during the current study.
