# Supplementary material for: Skin color reporting in squamous cell carcinoma-related randomized controlled trials in top dermatology journals: a systematic review
Source: Arch Dermatol Res. 2024 Mar 30;316(4):115. doi: 10.1007/s00403-024-02843-2 (PMC10981586; doi:10.1007/s00403-024-02843-2)
Supplement: Supplementary file 1 — Supplementary file1 (DOCX 21 KB) [file 403_2024_2843_MOESM1_ESM.docx]

# Purpose: To determine if our manuscript, if published, would be the first to publish the identification of skin color in squamous cell carcinoma studies amongst the top dermatology journals.

# Search Strategy: Databases were queried with the search terms in the table below.

# The abstracts were selected based on our inclusion criteria and were analyzed thoroughly to determine if they discussed randomized control trials involving squamous cell carcinoma (SCC). When the abstracts were inconclusive, the full article was read to inquire more details on the randomized control trials.

# Inclusion Criteria: Randomized control trial of SCC, Human patients, written in English

# Exclusion Criteria: No patients involved, full text unavailable

| **Database** | **Search Terms** | **Dates Queried** |
| --- | --- | --- |
| PubMed | 1. ["J Am Acad Dermatol” AND "Squamous Cell Carcinoma”]    2. [“JAMA Dermatology" AND "Squamous Cell Carcinoma”]  3. [“Br J Dermatol” AND ”Squamous Cell Carcinoma”]  4. [“J Eur Acad Dermatol Venereol” AND “Squamous Cell Carcinoma”]  5. [“J Invest Dermatol” AND “Squamous Cell Carcinoma”]  6. [“Contact Dermatitis” AND “Squamous Cell Carcinoma”]  7. [”Am J Clin Dermatol” AND ”Squamous Cell Carcinoma”]  8. [“J Dermatol Sci” AND ”Squamous Cell Carcinoma”]  9. [“Dermatology” AND ”Squamous Cell Carcinoma”]  10. [“Dermatitis” AND ”Squamous Cell Carcinoma”] | Any Time |

**Results:** We were able to find a couple articles that involved randomized control trials of squamous cell carcinoma in people with skin of color (SOC) and a Fitzpatrick scale greater than III but the vast majority of these trials were emphasized in white patients. Upon reviewing the 39 articles that fit our inclusion criteria, we found that only 23 of those articles reported skin color. Skin color reporting can be beneficial as the most common type of skin cancer for patients with SOC is SCC and the symptoms may present differently with varying skin colors (7). This search validates that if published, our systematic review would be a valuable addition to dermatological literature that often overlooks how important skin reporting can be.
